# 

**Published:** 1987-08

**Authors:** 


**Editor**

Mr M. G. Wilson

*40 Coombe Lane, Bristol BS9 2BJ*

*Tel: 0272-682320*

**Assistant Editor**

Dr P. R. Goddard

**Editorial Committee**

Mr B. P. Jones (Secretary),

*Medical Librarian, Bristol University*

Dr J. L. Burton

**Correspondents**

Dr A. E. Adam (Taunton)

Dr J. D. Davies

Dr J. Jancar

Professor D. J. Pereira Gray (Exeter)

Dr H. G. Mather

Professor G. M. Stirratt

Mr C. Teasdale (Plymouth)

Dr J. L. G. Thomson

Dr M. J. Whitfield

Professor G. K. Wilcock

Professor R. C. N. Williamson

Dr D. W. Wright

**BRISTOL MEDICO-CHIRURGICAL SOCIETY**

**President**

Mr H. B. Griffith

**Secretary**

Mr R. N. Baird,

*23 Old Sneed Park, Bristol BS9 1RG*

**Treasurer**

Dr N. C. Tricks,

*The Mill, Wrington, Bristol*

**Published by**

**Hermiston Publications,**

*BMA Scottish House,*

*7 Drumsheugh Gardens,*

*Edinburgh EH3 7QP*

**Production Manager**

June Macnab

**Typesetting by**

*Avon Communications Ltd.,*

*Avon House, Nailsea, Bristol BS19 2DJ*

**Printed by**

*Scottish County Press, Sherwood Industrial Estate,*

*Bonnyrigg, Midlothian*

Established 1883

### BRISTOL MEDICO-CHIRURGICAL JOURNAL

**AUGUST 1987**
**Volume 102 (iii)**

Published quarterly and distributed free of charge on request to doctors in the South West of England.
Overseas subscription £12 per annum.

What I know remains unknown, unless by me to others shown.
*Lucilius (180–102 BC)*

### BRISTOL MEDICINE

**Bristol Medicine acknowledges with gratitude the support it receives from Pfizer Ltd.**

**NOTICE TO CONTRIBUTORS**
Contributions are invited especially from West of England authors on a variety of subjects, e.g. Original articles relating to the practice of medicine, reports on the contemporary medical scene, obituaries, historical accounts, recollections of colleagues and events worthy to be remembered and liable to be forgotten. An article of 2,000 words with 2 illustrations will occupy 2 pages and this is a suggested, though by no means an obligatory length. Articles are preferred in the form proposed by the International Committee of Medical Editors (Vancouver System)—pamphlet obtainable from Editor of BMJ., BMA. House, Tavistock Square, London, WC1H 9JR.

